# Genetic and physiological determinants of lettuce partial resistance to Impatiens necrotic spot virus

**DOI:** 10.3389/fpls.2023.1163683

**Published:** 2023-06-08

**Authors:** Ivan Simko, Daniel K. Hasegawa, Hui Peng, Rebecca Zhao

**Affiliations:** ^1^ Crop Improvement and Protection Research Unit, Agricultural Research Service, U.S. Department of Agriculture, Salinas, CA, United States; ^2^ Horticultural Sciences Department, Everglades Research and Education Center, University of Florida, Belle Glade, FL, United States

**Keywords:** lettuce, tospovirus, genome-wide association study, western flower thrips, plant development, anthocyanins, chlorophyll, thrips feeding damage

## Abstract

**Introduction:**

Impatiens necrotic spot virus (INSV) is a major pathogen currently threatening lettuce (*Lactuca sativa* L.) production in the coastal areas of California. The virus is transmitted by the western flower thrips (Frankliniella occidentalis Pergande).

**Methods:**

We have tested a diversity panel of almost 500 lettuce accessions for disease incidence (DI) in 12 field experiments performed over 7 years. This set of accessions was also assessed for thrips feeding damage (TFD), the rate of plant development (PD), and the content of chlorophyll (SPAD) and anthocyanins (ACI) to determine their effect on resistance to INSV. In addition, recombinant inbred lines from two biparental mapping populations were also evaluated for DI in field experiments.

**Results:**

The mean DI in 14 field experiments ranged from 2.1% to 70.4%. A highly significant difference in DI was observed among the tested accessions, with the overall lowest DI detected in the red color cultivars, Outredgeous Selection, Red Splash Cos, Infantry, Sweet Valentine, Annapolis, and Velvet. Multiple linear regression models revealed a small but significant effect (*p* < 0.005) of the four analyzed determinants on DI. Accessions with lower DI values had slower plant development (PD, *r* = 0.352), higher ACI content (*r* = −0.284), lower TFD (*r* = 0.198), and lower SPAD content (*r* = 0.125). A genome-wide association study revealed 13 QTLs for DI located on eight out of the nine lettuce chromosomes (the exception was chr. 8). The most frequently detected QTL (*qINSV2.1*) was located on chr. 2. Several of the QTLs for DI were in the same genomic areas as QTLs for PD, ACI, and SPAD. Additional three QTLs for DI on chr. 5 and 8 were identified using linkage mapping performed on two biparental mapping populations.

**Conclusions:**

The work highlights the genetic basis of partial resistance to INSV and reveals the relationship between resistance, the host physiology, and the thrips vector. Results of this study are an important steppingstone toward developing cultivars with increased resistance against INSV.

## Introduction

Lettuce (*Lactuca sativa*) is a high-value leafy vegetable. Over 90% of U.S. production is grown predominantly in the states of California and Arizona, with over 60% of California’s production occurring in Monterey County (California Department of Food and Agriculture, 2021). Production within the region is valued at over $1 billion annually, with a broad range of cultivars grown across more than 40,000 ha to supply domestic and international markets, including whole-head lettuce or hearts, baby-leaf lettuce, spring mix varieties, or processing types for salad mixes (California Department of Food and Agriculture, 2021; Monterey County Agricultural Commissioner’s Office, 2021).

**Figure 1:**
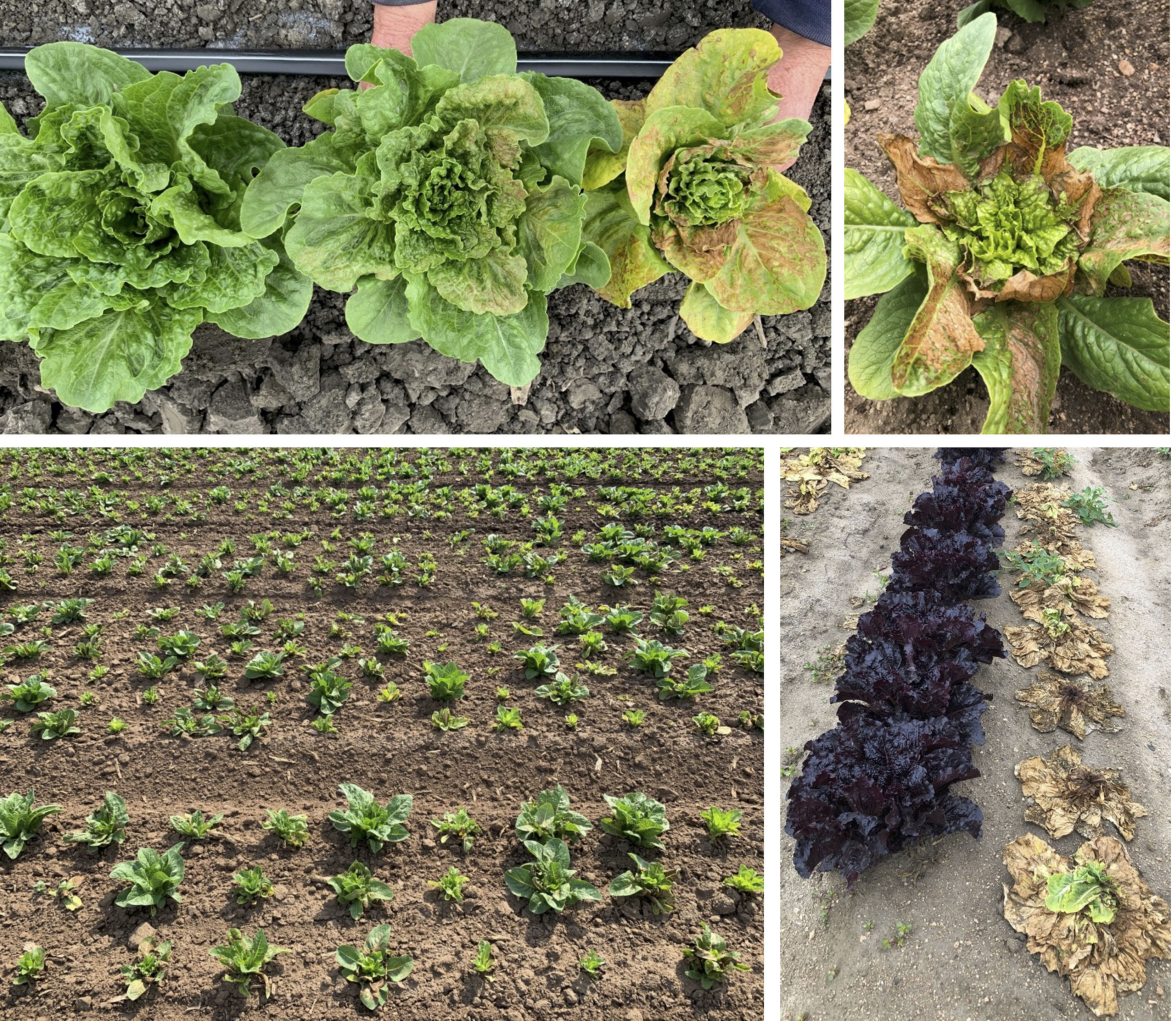
Symptoms of Impatiens necrotic spot virus (INSV) infection on field-grown lettuce plants. Top row: INSV symptoms on individual plants. Bottom row: fields with a high disease incidence. Note the difference in the disease incidence between dark-red and green accessions; dead plants are likely infected both with INSV and *Pythium uncinulatum*.

Impatiens necrotic spot virus (INSV; family *Tospoviridae*, genus *Orthotospovirus*) that is transmitted by the western flower thrips, *Frankliniella occidentalis* (suborder Terebrantia, family Thripidae, subfamily Thripinae), was first documented in lettuce in Monterey County in 2006 (Koike et al., 2008). Recently, INSV has emerged as a primary concern for the lettuce industry, accounting for the loss of hundreds of millions of U.S. dollars (Hasegawa and Del Pozo-Valdivia, 2022). Furthermore, the virus has been recently spread to the desert regions of Southern California and Arizona (Hasegawa et al., 2022), which, together, is the second largest lettuce-producing region in the United States (Simko et al., 2014). Until about 2017, the disease typically appeared in the Salinas Valley only later in the growing season (fall production) leaving earlier plantings almost unaffected (Simko, personal observation and personal communications from local growers). However, since 2019, the disease has been frequently observed as early as late February with incidences sustaining throughout the remainder of the long lettuce-growing season in the Salinas Valley (plantings occur between December and August, harvest between March and November) (Simko et al., 2014). Leaves of infected plants show brown to dark-brown spots, distorted and/or stunted growth, and necrosis, making them unmarketable (4). Plants infected during the early stages of development or those severely infected at later developmental stages usually die (**Figure 1**). Cultivars of all horticultural lettuce types are susceptible to INSV (Simko et al., 2018).

Viruses within the genus *Orthotospovirus* (family *Tospoviridae*), including INSV and tomato spotted wilt virus (TSWV), infect hundreds of host plant species worldwide, many of which are economically important food and horticultural crops (Pappu et al., 2009). Orthotospoviruses are enveloped and single-stranded RNA viruses with a genome that is organized into three segments. The large segment is negative-sense, while the medium and small segments are ambisense in orientation. The six proteins that are translated include the L protein, an RNA-dependent RNA polymerase; nucleocapsid protein (N), which encapsidates the RNA genome; NSs protein, an RNA silencing suppressor in plant and insect cells; a movement protein, NSs; and Gn/Gc glycoproteins, which are found on the surface of the virion (Oliver and Whitfield, 2016; Rotenberg and Whitfield, 2018).

Western flower thrips is globally distributed and an important vector for orthotospoviruses, including INSV and TSWV (Rotenberg et al., 2020). Thrips are especially difficult to manage due to their small size (1–2 mm), demonstrated resistance to insecticides, and ability to occupy an extensive host range of plant species that can support their growth and reproduction (Rotenberg et al., 2020). There are limited chemical options for managing thrips effectively in California lettuce, and due to their high efficiency in virus transmission, management of INSV remains particularly challenging. To create new management tools, breeding efforts are underway to develop lettuce cultivars that are resistant to INSV (Simko et al., 2018). The objectives of the current study were to identify accessions with a partial resistance to INSV, to map loci associated with INSV resistance, and to determine physiological factors contributing to the variability in resistance observed among lettuce accessions.

## Material and methods

### Plant material

Lettuce is a self-fertilizing diploid species (2*n* = 2*x* = 18) from the family *Compositae* (*Asteraceae*). A diversity panel used for the genome-wide association study (GWAS) comprised 496 lettuce accessions belonging to romaine, Batavia, butterhead, iceberg, Latin, and leaf horticultural types and three *L. serriola* accessions. In addition, two accessions of *L. indica* were included in the diversity panel for a phenotypic comparison but were not used in the GWAS. This diversity set was described in detail in previous studies that focused on mapping quantitative trait loci (QTLs) for plant development, postharvest quality (Sthapit Kandel et al., 2020), and resistance to yellow spot malady (Peng et al., 2022), downy mildew (Simko et al., 2022a) and bacterial leaf spot (Sthapit Kandel et al., 2022). Two biparental mapping populations used for linkage mapping consisted of 88 (GR × Sal) and 160 (Ice × PI) F_7_ recombinant inbred lines (RILs). These populations were developed by crossing cvs. Grand Rapids and Salinas (GR × Sal) and cv. Iceberg and PI 491224 (Ice × PI). Both populations were previously used to identify QTLs for resistance to downy mildew (Parra et al., 2021). All three mapping populations were genotyped with single-nucleotide polymorphism (SNP) markers anchored to the cv. Salinas reference genome v.8 (Zhang et al., 2017). After filtering, 4,615 high-quality SNP markers were used for mapping in the diversity panel and 751 and 1,635 markers in the GR × Sal and Ice × PI population, respectively.

### Field assessment of resistance to Impatiens necrotic spot virus and thrips damage

INSV incidence was evaluated on mature plants grown in 14 field experiments (12 for GWAS and 2 for linkage mapping) in the Salinas Valley, California between 2016 and 2022 (**Table 1**). Field experiments were set up with three replications using either the Randomized Complete Block Design (RCBD) or the Augmented Randomized Complete Block Design (ARCBD) with 39 control accessions. Approximately 20–30 plants per plot were evaluated for disease symptoms at harvest maturity, though weekly evaluations were carried out to ascertain that plants were infected by INSV. Disease incidence was expressed as the percentage of symptomatic plants out of the total number of plants (DI%) in a plot. The presence of INSV in randomly selected symptomatic plants was confirmed by immunostrips (Agdia, Elkhart, Indiana, USA) or enzyme-linked immunosorbent assay (ELISA) (Simko et al., 2018). The disease incidence was recorded from individual plots and data across all three replications in RCBD were averaged for each accession. To account for possible differences among field blocks in ARCBD, replicated control accessions were used to calculate least-square means (LS means of DI%). Because average DI% values (or LS means of DI%) from all experiments showed skewed distribution when tested with the Anderson–Darling test (**Table 1**), data from each experiment were transformed using bestNormalize v. 1.8.3 R package (Peterson and Cavanaugh, 2020). Transformed data (normalized and standardized values with mean ~ 0 and standard deviation ~ 1) from 12 experiments with the diversity panel were averaged across all experiments to calculate an overall level of resistance for each accession (INSVx).

**Table 1:** Impatiens necrotic spot virus (INSV) incidence at 14 field experiments used in the genome-wide association studies (GWASs) and linkage mapping.

Experiment^a^	No. of phenotyped accessions	INSV incidence (%)					
		Minimum^d^	Maximum^d^	Mean ± St. Err.	Median	Range	A^2^
I16-S1	446	0.0	25.0	2.6 ± 0.2	0.0	25.0	43.1
I18-S1	495	-3.5	102.6	32.0 ± 1.0	30.0	106.1	5.0
I19-S1	491	-5.9	97.7	16.9 ± 0.8	13.3	103.7	17.8
I19-S2	490	-20.7	116.8	47.9 ± 1.4	46.8	137.5	5.9
I19-S3	489	-6.7	106.9	40.8 ± 1.4	36.7	113.6	7.3
I20-B1	494	-1.2	52.9	7.5 ± 0.4	4.0	43.1	25.9
I20-B2	489	-4.9	104.7	14.2 ± 0.7	10.9	109.6	12.8
I20-B3	491	-1.5	65.2	6.6 ± 0.5	1.8	66.7	34.3
I20-C1	490	-1.2	48.8	2.1 ± 0.3	0.5	50.0	75.9
I21-B1	498	0.0	60.0	11.9 ± 0.4	10.1	60.0	9.1
I21-C1	497	-1.1	80.8	5.4 ± 0.4	0.8	81.9	49.1
I22-S1	498	0.0	100.0	70.4 ± 1.2	80.0	100.0	18.5
GR × Sal^b^	88^c^	1.2	62.4	15.3 ± 1.4	12.1	61.2	3.1
Ice × PI^b^	160^c^	1.2	62.4	20.2 ± 0.9	19.6	61.2	0.9

a Names of GWAS experiments use the following code: ‘I’ stands for ‘INSV’; two-digit number indicates the year of the experiment from 2016 to 2022; the letter after dash specifies field location, ‘B’, Salinas field B; ‘C’, Salinas field C; ‘S’, Salinas Spence field, and the last numeral indicates the order of the experiments performed within the same year in the same field.

b Biparental mapping population used in linkage mapping.

c Due to incomplete genotypic information, only 86 and 124 of the RILs were used in linkage mapping, respectively.

d Due to the adjustments for covariates (replicated controls in the Augmented Randomized Complete Block Design), the minimum and maximum of LSmeans values may be somewhat outside of the original range (0%– 100%).

A^2^ values for the Anderson–Darling test. All test values exceeded the critical values (at p < 0.05), thus rejecting the normality of the distribution.

To evaluate thrips damage, assessments were made on plants from the I19-S1 experiment (**Table 1**). Three lettuce plants were randomly collected from each plot and brought to the laboratory 34 days after planting to evaluate the damage caused by thrips. A single, first true leaf was detached from each plant and scored for thrips damage, using a 0–5 rating scale. The rating scale for thrips feeding damage (TFD) was as follows: 0 = no scars, 1 = 1–25 scars, 2 = 26–50 scars, 3 = 51–75 scars, 4 = 76–100 scars, 5 = >100 scars. Data across all plants and replications were averaged for each accession. Average values of TFD were transformed using bestNormalize v. 1.8.3 R package (Peterson and Cavanaugh, 2020) before statistical analyses.

### Evaluations of plant development and the content of pigments

Accessions included in the diversity panel were previously evaluated for the rate of their development in five field experiments using a 1–7 scale (1 = rosette, 2 = bolting, 3 = visible buds, 4 = expanded inflorescence, 5 = flowering, 6 = majority flowered, and 7 = mature seeds) (Sthapit Kandel et al., 2020). Since then, the same diversity panel was evaluated for the rate of development in other eight field experiments, bringing the total number of evaluated experiments located in the Salinas Valley to 13. Data from each experiment were transformed using bestNormalize v. 1.8.3 R package (Peterson and Cavanaugh, 2020) and combined into a single dataset (PDx) by averaging transformed values across all experiments. The PDx values that represent an average rate of plant development in the Salinas Valley were subsequently used in all statistical analyses.

The content of chlorophyll and anthocyanins was assessed on plants from the I22-S1field trial. Three largest leaves were evaluated on three randomly selected plants of each accession at harvest maturity. The contents of chlorophyll and anthocyanins were determined at the position of ca. 2 cm from a leaf tip (avoiding leaf veins) using SPAD-502 (Konica Minolta Sensing Inc., Tokyo, Japan) and ACM-200 plus (Opti-Sciences, Hudson, New Hampshire, USA) meters, respectively. The average values of pigments from each accession were transformed as previously recommended (Simko, 2020): the square root transformation of SPAD values for chlorophyll (SPAD-Sqrt) and the binary logarithm transformation of ACI values for anthocyanins (ACI-Lb). Transformed data were used in subsequent statistical analyses.

### Statistical analyses

The relationship between sets of data was evaluated using Pearson’s linear correlation coefficient (*r*) and Spearman’s rank correlation coefficient (*ρ*). To test the effect of multiple independent variables on INSV DI%, the multiple linear regression model was applied. All statistical analyses, including the Anderson–Darling test and calculations of LS means mentioned above, were performed using JMP Pro v. 17.0.0 (SAS Institute, Cary, North Carolina, USA). Data transformations were carried out using the bestNormalize v. 1.8.3 R package (Peterson and Cavanaugh, 2020).

### Association mapping, linkage mapping, and quantitative trait locus nomenclature

GWAS on data from the diversity panel was performed with the FarmCPU software that controls false-positive associations by preventing model overfitting (Liu et al., 2016). The threshold for significant QTL was set at genome-wide α = 0.05 using the Bonferroni correction. Linkage mapping in two biparental mapping populations was carried out with QGene v. 4.4.0 software (Joehanes and Nelson, 2008) using the composite interval mapping approach and an automated, forward selection of cofactors. The threshold for significant QTL (at genome-wide α = 0.05) was determined through permutations with 1,000 iterations.

The naming of QTLs in this paper follows the previously proposed nomenclature (Simko et al., 2013) starting with the letter ‘*q*’ (QTL), followed by the trait acronym (INSV, SPAD, or ACI), the chromosome (chr.) number where the QTL was located, and the QTL number starting with 1 and increasing as needed (e.g., *qINSV5.2* is the second QTL for resistance to INSV detected on chr. 5). A QTL located in the same genomic region as a previously described QTL for the corresponding trait was given the name of the original QTL, regardless of the naming nomenclature.

## Results

### Phenotypic relationship of pigments, plant development, and thrips with Impatiens necrotic spot virus resistance

Tested accessions showed a range of colors, visually appearing as light green, green, dark green, light red, red, and dark red. Because lettuce color is mostly moderated by the content of chlorophyll and anthocyanins (Simko, 2020), the content of these two pigments was quantified in leaf tissue. The content of chlorophyll (SPAD-Sqrt) ranged from 4.66 (cv. Blush Butter Cos) to 8.16 (cv. Red Cos) with a mean value of 6.75 (**Supplemental Table 1**). The content of anthocyanins (ACI-Lb) ranged from 1.81 (PI 278064) to 9.21 (cv. Annapolis) with a mean value of 3.18. The average rate of plant development (PDx) ranged from the slowest −1.16 for PI 234624 to the most rapid 2.43 for PI 278097 (**Supplemental Table 1**). The TFD rating calculated from the number of scars on a leaf ranged (before transformation) from 0.40 (PI 226514) to 4.80 (cv. Freckles) with a mean value of 1.85 (**Supplemental Table 1**).

The average INSV incidence at 14 field experiments ranged from 2.1% (I20-C1) to 70.4% (I22-S1) (**Table 1**). Because data at all experiments showed a skewed distribution (left-tailed in I22-S1, right-tailed in all other experiments), the original DI% data were transformed using the bestNormalize R package (Peterson and Cavanaugh, 2020) before analyses. Among 498 tested accessions, the overall lowest DI was observed for SAL016 (INSVx = -1.27), cvs. Outredgeous Selection (-1.17), Red Splash Cos (-1.13), Infantry (-1.11), Sweet Valentine (-1.06), Annapolis 4x (-1.01), and Velvet (-1.00) (**Supplemental Table 1**). SAL016 is *L. indica* accession that was not used in GWAS. All other accessions in this group were light red to dark red (ACI-Lb from 4.00 to 8.02, SPAD-Sqrt from 5.70 to 6.56). The overall most susceptible accessions were cvs. Balady Banha (INSVx = 1.39), Balady Barrage (1.22), PI 167128 (1.08), PI 491216 (1.08), PI 342521 (1.04), and PI 268405 (1.02). All these accessions were green (ACI-Lb from 2.38 to 3.29, SPAD-Sqrt from 6.06 to 6.94).

Pearson’s linear correlation coefficient between pairs of experiments used in the GWAS ranged from *r* = -0.070 (*p* = 0.121) between I20-C1 and I21-C1 to *r* = 0.383 (*p* < 0.0001) between I18-S1 and I22-S1 (**Supplemental Figure 1**). Overall, 40 out of 66 pairwise comparisons showed a positive and significant (*p* < 0.05) correlation between experiments. Highly similar results were obtained using Spearman’s rank correlation coefficient. The average disease incidence across all experiments (INSVx) displayed a significant (*p* < 0.0001), positive correlation with all individual datasets (*r* from 0.200 with I20-C1 to 0.522 with I22-S1, *ρ* from 0.288 with I20-C1 to 0.554 with I22-S1) (**Supplemental Figure 1**).

No significant correlation was detected between TFD and the content of anthocyanins (*r* = 0.030, *p* = 0.510) or chlorophyll (*r* = -0.030, *p* = 0.512). A weak (*r* = 0.093) but significant (*p* = 0.040) correlation was found, however, between the plant developmental rate and TFD, indicating that accessions with more rapid development were more damaged by thrips. These results were confirmed when all three factors (ACI-Lb, SPAD-Sqrt, and PDx) were used together in the multiple linear regression model where TFD was used as a dependent variable.

**Figure 2:**
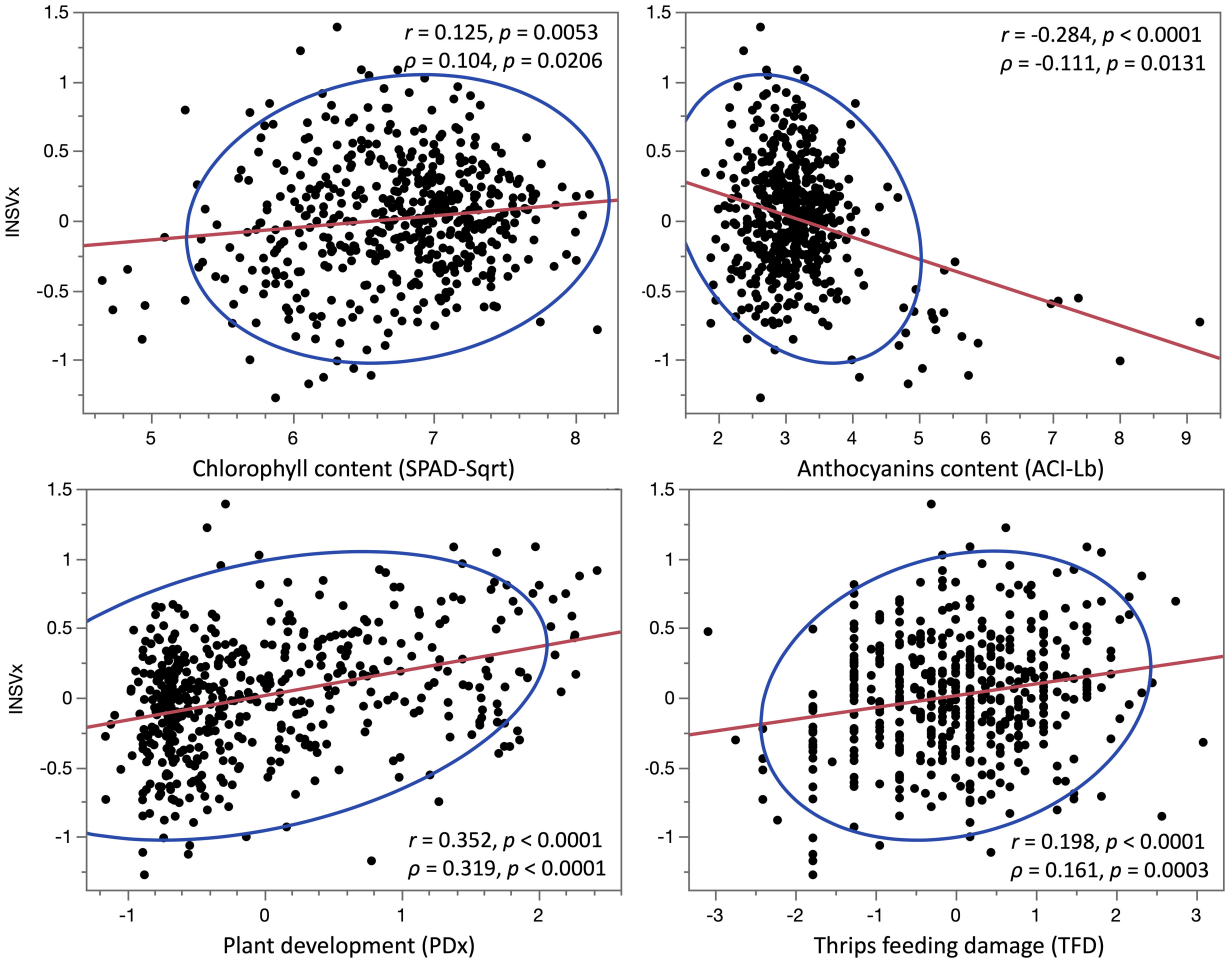
Correlations between four phenotypic variables and lettuce partial resistance to INSV. Values of the Pearson’s correlation coefficient (*r*) and the Spearman’s rank correlation coefficient (*ρ*) are shown for the relationship between INSV incidence (INSVx) and the content of chlorophyll (SPAD-Sqrt), anthocyanins (ACI-Lb), plant development (PDx), and thrips feeding damage (TFD). Higher values of variables indicate a higher INSV incidence (INSVx), higher contents of pigments (SPAD-Sqrt and ACI-Lb), more rapid plant development or earlier bolting (PDx), and more scars on a leaf caused by thrips feeding (TFD). The red lines indicate trends between variables, while the blue lines define the 95% confidence ellipses.

When the contents of anthocyanins (ACI-Lb) and chlorophyll (SPAD-Sqrt), plant development (PDx), and TFD were compared to INSV incidence at 12 field experiments, significant correlations (*p* < 0.05) were found with PDx at 11 experiments, ACI-Lb at 8 experiments, TFD at 6 experiments, and SPAD-Sqrt at 2 experiments (**Table 2**). The multiple linear regression model confirmed most of the significant results (nine for PDx and all eight for ACI-Lb and six for TFD), plus revealed an additional significant relationship with SPAD-Sqrt at five more experiments (seven experiments in total). All four variables had a highly significant relationship (*p* < 0.001) with an average DI% (INSVx) both when analyzed individually (**Table 2**, **Figure 2**) or in a multiple linear regression model (**Table 2**). Matching results were obtained when *p*-values from multiple linear regressions were combined across 12 field experiments using Fisher’s method (*p* < 0.001). Overall, lower INSV incidence associated the most with a slower plant development (PDx), followed by higher anthocyanin content (ACI-Lb), lower chlorophyll content (SPAD-Sqrt), and less TFD.

**Table 2:** Relationship between INSV incidence at 12 field experiments and a combined dataset (INSVx) with the content of anthocyanins (ACI-Lb), chlorophyll (SPAD-Sqrt), plant development (PDx), and thrips feeding damage (TFD).

Experiment	ACI-Lb			SPAD-Sqrt			PDx			TFD			MLR model	
	*r*	*p*	*p_m_*	*r*	*p*	*p_m_*	*r*	*p*	*p_m_*	*r*	*p*	*p_m_*	*R^2^%*	*p_m_*
I16-S1	-0.026	0.580	0.613	0.013	0.777	0.590	0.106	0.026	0.051	0.113	0.018	0.030	2.3	0.040
I18-S1	-0.293	0.000	0.000	0.035	0.439	0.003	0.224	0.000	0.000	0.081	0.073	0.069	14.0	0.000
I19-S1	-0.058	0.199	0.416	-0.037	0.413	0.904	0.184	0.000	0.000	0.181	0.000	0.000	6.2	0.000
I19-S2	-0.152	0.001	0.000	0.081	0.074	0.001	0.172	0.000	0.001	0.199	0.000	0.000	10.1	0.000
I19-S3	-0.172	0.000	0.000	0.153	0.000	0.000	0.227	0.000	0.000	0.105	0.020	0.022	12.7	0.000
I20-B1	-0.132	0.003	0.002	0.036	0.418	0.050	0.138	0.002	0.011	0.121	0.008	0.008	5.2	0.000
I20-B2	-0.002	0.958	0.827	0.083	0.068	0.035	0.129	0.004	0.005	0.104	0.023	0.035	3.5	0.002
I20-B3	-0.099	0.029	0.018	0.082	0.069	0.010	0.104	0.022	0.027	-0.035	0.444	0.419	3.3	0.003
I20-C1	-0.107	0.018	0.034	-0.024	0.597	0.900	0.050	0.272	0.433	-0.018	0.698	0.691	1.2	0.197
I21-B1	-0.100	0.025	0.005	0.132	0.003	0.000	0.148	0.001	0.002	0.074	0.104	0.116	6.3	0.000
I21-C1	-0.067	0.133	0.355	0.014	0.754	0.200	0.238	0.000	0.000	0.049	0.279	0.494	6.1	0.000
I22-S1	-0.276	0.000	0.000	0.082	0.067	0.000	0.111	0.014	0.080	0.055	0.222	0.149	11.1	0.000
INSVx	-0.284	0.000	0.000	0.125	0.005	0.000	0.352	0.000	0.000	0.198	0.000	0.000	26.5	0.000

r, Pearson’s linear correlation coefficient; p, p-value of the Pearson’s correlation coefficient; p_m_, p-value for a given variable from the multiple linear regression model that uses all four variables together; MLR model, multiple linear regression model; R^2^%, percent of variation explained by the model.

p-values of 0.000 are smaller than 0.0005.

### Quantitative trait loci for chlorophyll and anthocyanins content detected through genome-wide association study

Previously performed analyses of population structure revealed a possible presence of three main subgroups within the diversity panel, with a potential further division into six to eight subgroups (Sthapit Kandel et al., 2020; Peng et al., 2022; Simko et al., 2022a). GWAS analysis carried out on SPAD-Sqrt and ACI-Lb data revealed four QTLs associated with the content of chlorophyll and 12 QTLs for anthocyanins (**Table 3**). QTLs for chlorophyll content were located on chr. 3, 4 (two QTLs), and 8, while those for anthocyanins were found on chr. 2, 3, 4 (three QTLs), 5 (two QTLs), 7 (two QTLs), 8, and 9 (two QTLs). One of the QTLs for the content of chlorophyll (*qLG4.1*) was previously detected in multiple mapping populations (Kwon et al., 2013; Simko et al., 2016; Mamo et al., 2019; Parra et al., 2021, Simko et al., unpublished results), while the other three QTLs appeared to be located at the genomic regions not previously associated with the content of chlorophyll. Out of 12 QTLs for the content of anthocyanins, 7 (*qRLC2.1*, *RLL3*, *RLL2*, *qRLC5.2*, *qRLC7.2*, *RLL4*, and *ANS*) were found at or near QTLs and genes for red leaf color or anthocyanin content detected in previous studies (Kwon et al., 2013; Mamo et al., 2019; Gurdon et al., 2021; Simko et al., 2022b, Simko et al., unpublished results, Zhang et al., 2017; Su et al., 2020; Wei et al., 2021; Wada et al., 2022), while the remaining five QTLs were new.

**Table 3:** Quantitative trait loci (QTLs) for the content of chlorophyll (SPAD-Sqrt) and anthocyanins (ACI-Lb) mapped in the diversity panel of 496 accessions.

QTL/Gene^a^	Trait	Chr.	Position	Bin	-Log_10_(*p*)	Note and reference
*qSPAD3.1*	SPAD-Sqrt	3	107,109,728	3.11	5.0	New QTL
*qLG4.1*	SPAD-Sqrt	4	101,350,893	4.11	4.9	(Kwon et al., 2013; Simko et al., 2016; Mamo et al., 2019; Parra et al., 2021, Simko et al., unpublished results)
*qSPAD4.1*	SPAD-Sqrt	4	145,945,115	4.15	6.1	New QTL
*qSPAD8.1*	SPAD-Sqrt	8	159,751,796	8.16	5.5	New QTL
						
*qRLC2.1*	ACI-Lb	2	23,589,102	2.03	6.2	(Kwon et al., 2013, Simko et al., unpublished results)
*qACI3.1*	ACI-Lb	3	50,171,303	3.06	7.8	New QTL
*RLL3*	ACI-Lb	4	34,498,615	4.04	4.9	(Simko et al., unpublished results, Zhang et al., 2017; Su et al., 2020; Wada et al., 2022)
	ACI-Lb	4	49,412,612	4.05	6.7	
*qACI4.1*	ACI-Lb	4	214,512,362	4.22	7.6	New QTL
*qACI4.2*	ACI-Lb	4	269,983,062	4.27	5.5	New QTL
*RLL2*	ACI-Lb	5	80,705,220	5.09	5.4	(Mamo et al., 2019, Simko et al., unpublished results, Zhang et al., 2017; Su et al., 2020; Wada et al., 2022)
	ACI-Lb	5	83,165,802	5.09	10.8	
*qRLC5.2*	ACI-Lb	5	207,165,885	5.21	4.9	(Simko et al., unpublished results)
*qACI7.1*	ACI-Lb	7	132,477,227	7.14	5.3	Overlaps with the previously detected but unnamed QTL at the marker: RHCLSX699.b1_F08_3-OP3 (Kwon et al., 2013)
*qRLC7.2*	ACI-Lb	7	187,707,340	7.19	12.8	(Simko et al., unpublished results)
*qACI8.1*	ACI-Lb	8	201,378,657	8.21	5.2	Overlaps with the previously detected but unnamed QTL at the marker: CLS_S3_Contig2508-1-OP4 (Kwon et al., 2013)
*RLL4*	ACI-Lb	9	61,171,020	9.07	9.4	(Simko et al., unpublished results, Su et al., 2020; Wada et al., 2022)
*ANS*	ACI-Lb	9	120,881,337	9.13	7.8	(Kwon et al., 2013; Mamo et al., 2019; Gurdon et al., 2021; Simko et al., 2022b, Simko et al., unpublished results, Zhang et al., 2017; Su et al., 2020; Wei et al., 2021; Wada et al., 2022)

a If a QTL detected in the current study is located in the same or an adjacent bin as a QTL/gene detected in previous studies, it gets the same name as the previously detected QTL/gene.

### Quantitative trait loci for Impatiens necrotic spot virus incidence detected through genome-wide association study

**Figure 3:**
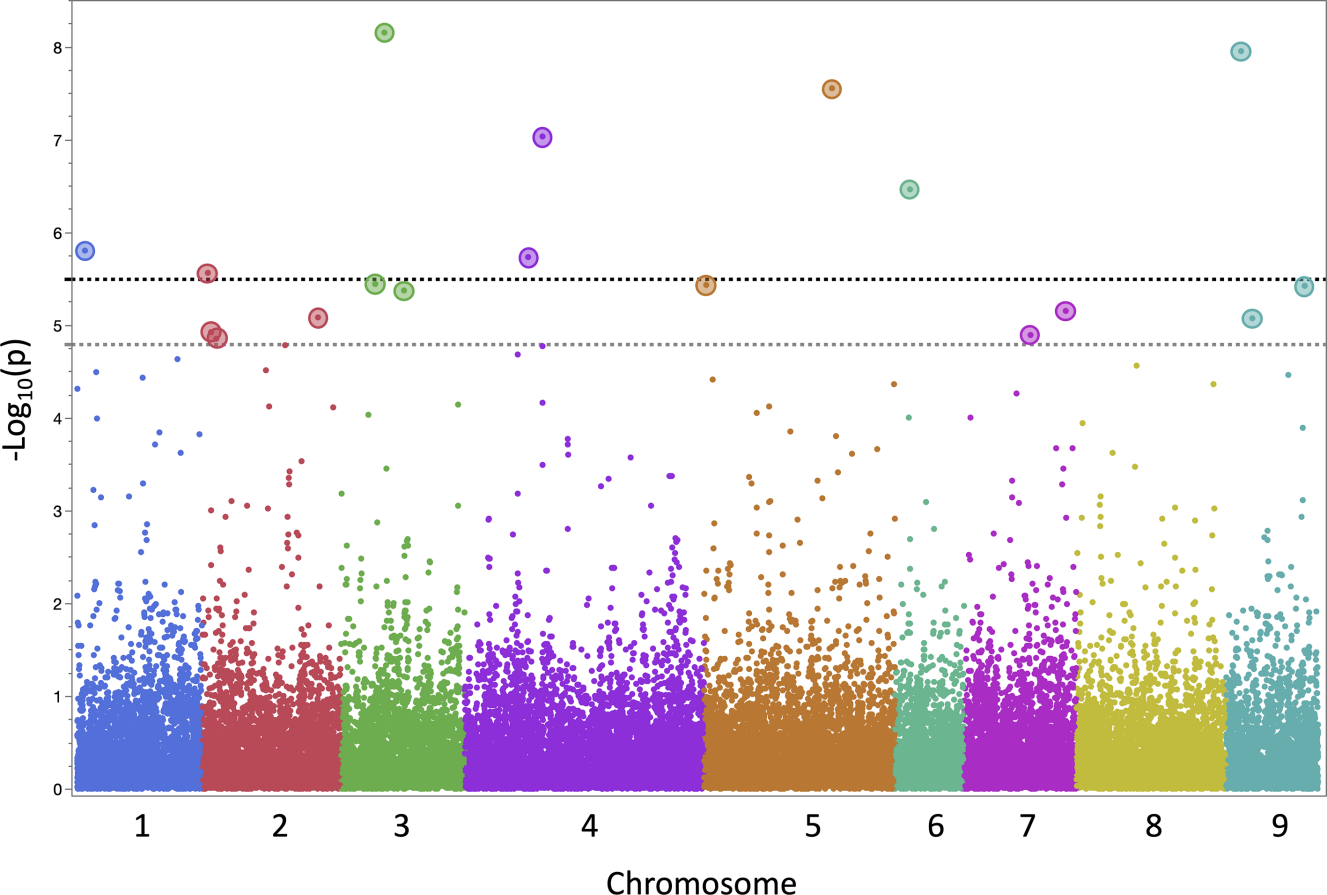
Manhattan plot of the results from genome-wide association (GWAS) mapping of quantitative trait loci (QTLs) for lettuce partial resistance to INSV. The plot shows a combined results from 13 datasets (12 field experiments plus a combined dataset). Two horizontal dashed lines indicate genome-wide significance threshold (*α* = 0.05 in gray, *α* = 0.01 in black) calculated using the Bonferroni correction. Single-nucleotide polymorphism markers significantly associated with resistance to INSV at *α* < 0.05 are emphasized by larger circles.

**Figure 4:**
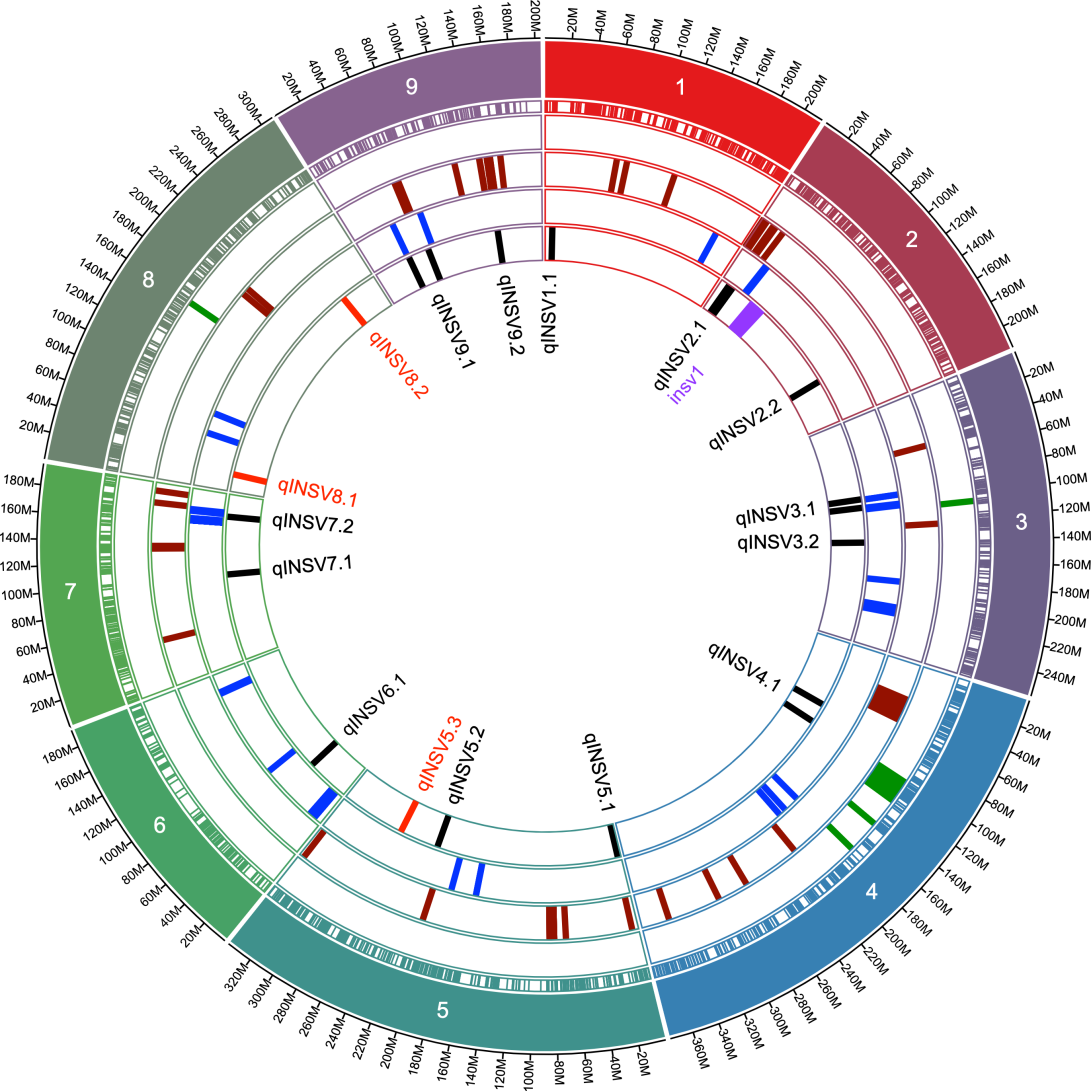
Circular graph showing the genomic locations of QTLs for resistance to INSV and their proximity to the loci for the content of chlorophyll, anthocyanins, and plant development. Concentric circles show (starting from inside) names of QTLs for resistance to INSV, locations of QTLs for resistance to INSV [current GWAS mapping results (in black), linkage mapping results (in red), previously described resistance locus (in purple) (Schut et al., 2016), plant development (in blue), content of anthocyanins or intensity of red leaf color (in maroon), content of chlorophyll or intensity of green color (in green), positions of molecular markers used in the current genome-wise association study, chromosome number, and the physical distance in mega base pairs].

Data from 13 datasets (12 field experiments plus INSVx dataset with combined data) were used to identify QTLs associated with INSV incidence. There were 13 QTLs detected on chr. 1 (*qINSV1.1*), 2 (*qINSV2.1*, *qINSV2.2*), 3 (*qINSV3.1*, *qINSV3.2*), 4 (*qINSV4.1*), 5 (*qINSV5.1*, *qINSV5.2*), 6 (*qINSV6.1*), 7 (*qINSV7.1*, *qINSV7.2*), and 9 (*qINSV9.1*, *qINSV9.2*) (**Table 4**, **Figures 3**, **4**). The most frequently detected QTLs were *qINSV2.1* found in three datasets (INSVx, I21-B1, and I21-S1), *qINSV3.1* in two datasets (I19-S1 and I21-C1), *qINSV4.1* in two datasets (INSVx and I21-C1), and *INSV9.1* in two datasets (I18-S1 and I19-S1). There were 9 out of 13 QTLs for resistance to INSV that appeared to be overlapping or to locate in the vicinity of QTLs or genes for pigments. QTLs for INSV resistance collocating with QTLs for chlorophyll content were *qINSV3.1* with *qSPAD3.1* at chr. 3, and *qINSV4.1* with *qLG4.1* at chr. 4. A possible collocation of QTLs for the resistance and anthocyanin content were detected at chr. 2 (*qINSV2.1* with *qRLC2.1*), chr. 3 (*qINSV3.2* with *GST*), chr. 5 (*qINSV5.1* with *qRLC5.1* and *qINSV5.2* with *qRLC5.2*), chr. 7 (*qINSV7.2* with *qRLC7.2*), and chr. 9 (*qINSV9.1* with *RLL4*, and *qINSV9.2* with *ANS*). QTLs for resistance to INSV also potentially overlapped with three QTLs for the rate of plant development that were previously detected using the accessions from this diversity panel (Sthapit Kandel et al., 2020; Rosental et al., 2021). The collocating QTLs were *qINSV3.1*, *qINSV7.2*, and *qINSV9.1* (**Figure 4**). No significant QTLs were detected for TFD.

**Table 4:** QTLs for partial resistance to INSV mapped in the diversity panel of 496 accessions and two biparental mapping populations.

QTL	Experiment	Chr.	Position	Bin	-Log_10_(*p*) or LOD^b^	Proximity to QTL/gene for chlorophyll or anthocyanins	Proximity to QTL for plant development
*qINSV1.1*	I18-S2	1	8,335,448	1.01	5.8		
*qINSV2.1*	INSVx	2	10,525,059	2.02	5.6	*qRLC2.1;* current study, (Kwon et al., 2013, Simko et al., unpublished results)	
	I21-B1	2	11,102,844	2.02	4.9		
	I21-S1	2	15,640,505	2.02	4.9		
*qINSV2.2*	I18-S1	2	165,187,508	2.17	5.1		
*qINSV3.1*	I19-S1	3	91,478,590	3.10	5.4	*qSPAD3.1;* current study	(Sthapit Kandel et al., 2020)
	I21-C1	3	101,406,161	3.11	8.2		
*qINSV3.2*	I21-B1	3	142,163,818	3.15	5.4	*GST* (may be too distant) (Zhang et al., 2017; Wada et al., 2022)	
*qINSV4.1*	INSVx	4	78,034,580	4.08	5.7	*qLG4.1;* (Kwon et al., 2013; Simko et al., 2016; Mamo et al., 2019; Parra et al., 2021, Simko et al., unpublished results)	
	I21-C1	4	101,036,448	4.11	7.0		
*qINSV5.1*	INSVx	5	3,722,751	5.01	5.4	*qRLC5.1;* (Simko et al., unpublished results)	
*qINSV5.2*	I19-S3	5	217,395,238	5.22	7.5	*qRLC5.2;* current study (Simko et al., unpublished results)	
*qINSV5.3*	Ice × PI^a^	5	263,509,090	5.27	6.1^b^		
*qINSV6.1*	INSVx	6	50,642,173	6.06	6.5		
*qINSV7.1*	INSVx	7	101,639,165	7.11	4.9		
*qINSV7.2*	I21-C1	7	169,235,060	7.17	5.1	*qRLC7.2;* current study (Simko et al., unpublished results)	(Sthapit Kandel et al., 2020)
*qINSV8.1*	GR × Sal^a^	8	20,129,380	8.03	6.3^b^		
*qINSV8.2*	GR × Sal^a^	8	266,269,492	8.27	7.5^b^		
*qINSV9.1*	I18-S1	9	43,462,427	9.05	8.0	*RLL4;* current study, (Simko et al., unpublished results, Su et al., 2020; Wada et al., 2022)	(Sthapit Kandel et al., 2020; Rosental et al., 2021)
	I19-S1	9	67,887,150	9.07	5.1		
*qINSV9.2*	I20-B1	9	151,974,224	9.16	5.4	*ANS*, current, (Kwon et al., 2013; Mamo et al., 2019; Gurdon et al., 2021; Simko et al., 2022b, Simko et al., unpublished results, Zhang et al., 2017; Su et al., 2020; Wei et al., 2021; Wada et al., 2022)	

a Biparental mapping population.

b The logarithm of the odds ratio is provided for the linkage mapping results using biparental mapping populations.

### Quantitative trait loci for Impatiens necrotic spot virus incidence detected through linkage mapping

**Figure 5:**
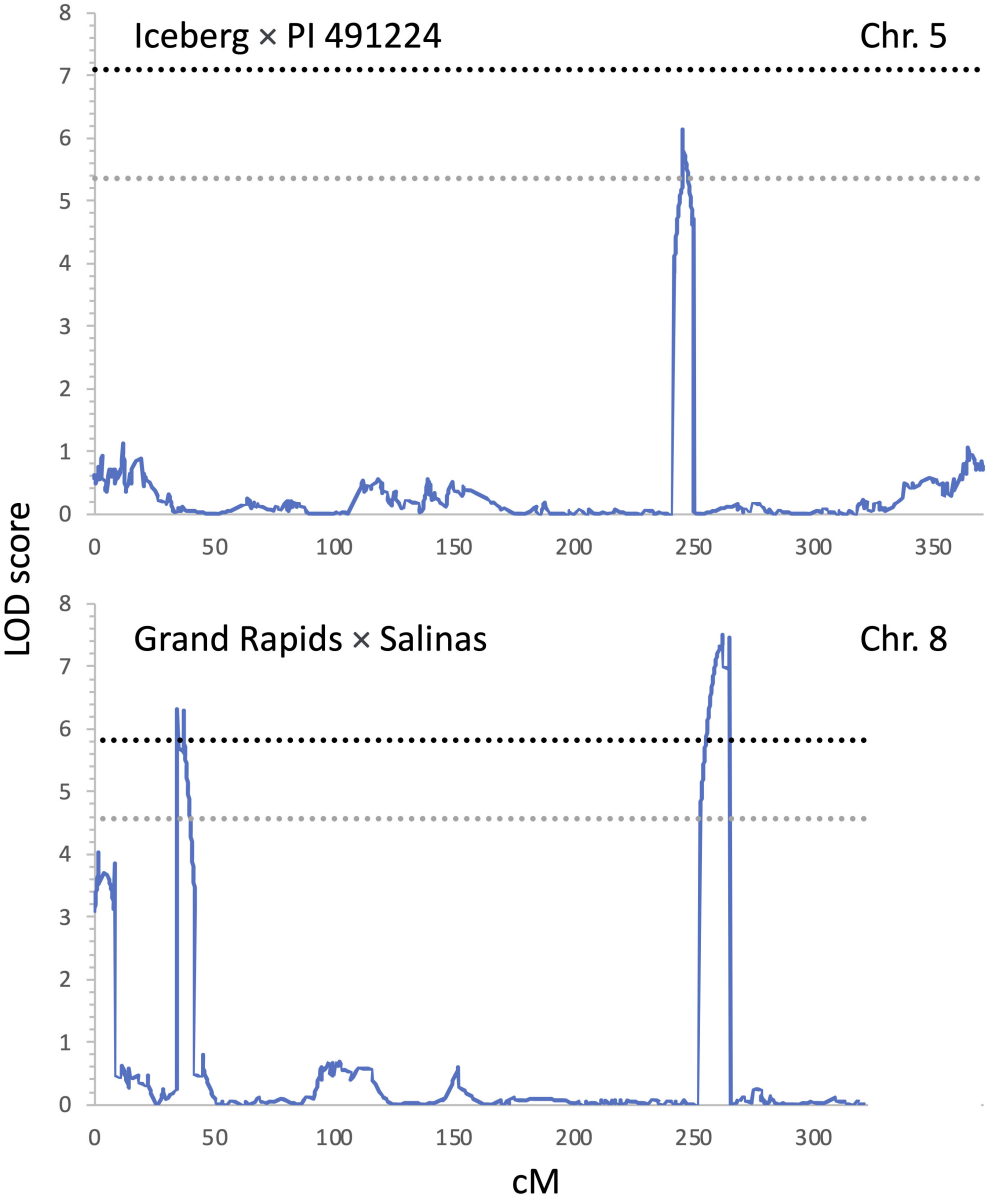
Positions of QTLs for resistance to INSV detected through linkage mapping at two biparental mapping populations. The top panel shows the QTL detected on chr. 5 in Iceberg × PI 491224 (Ice × PI) mapping population; the bottom panel shows two QTLs detected on chr. 8 in the Grand Rapids × Salinas (GR × Sal) mapping population. Significance thresholds for the logarithm of the odds scores are indicated with the gray (*α* = 0.05) and black (*α* = 0.01) horizontal dashed lines. Genetic distance is shown in centiMorgans (cM).

Two biparental mapping populations were used to map QTLs for resistance to INSV. The Ice × PI and GR × Sal populations consisted of 124 and 86 genotyped and phenotyped RILs, respectively. There was only a limited difference in the respective parental accessions in their rate of development and anthocyanin content. However, both populations segregated for the content of chlorophylls, the trait linked to *qLG4.1* (Simko et al., 2016; Parra et al., 2021) that was also detected in the current study (**Table 3**, **Figure 4**). Three QTLs linked with INSV incidence were mapped on chr. 5 (*qINSV5.3*) in the Ice × PI population and on chr. 8 (*qINSV8.1* and *qINSV8.2*) in the GR × Sal population (**Table 4**, **Figures 4**, **5**). None of the QTLs were located in the genomic regions associated with the content of chlorophyll, anthocyanins, plant development, or partial resistance to INSV detected in the diversity panel using GWAS.

## Discussion

In recent years, INSV has emerged as a primary threat to the lettuce industry in the Western United States (Hasegawa and Del Pozo-Valdivia, 2022; Hasegawa et al., 2022). Despite current efforts to improve monitoring and disease prevention, new management tools are needed. Surveys conducted in 10 commercial romaine lettuce fields in the Salinas Valley in 2008 and 2009 found that the average disease incidence was 5.7% (range from 0.5% to 27.0%) (Kuo et al., 2014). Later, data from 2015 and 2016, from six experimental fields with eight types of lettuce, found a similar average INSV incidence of 7.2% (range from 2.6% to 16.3%) (Simko et al., 2018). Our current data from 14 field experiments with a mix of lettuce types grown between 2016 and 2022 showed a substantially higher average disease incidence of 21.0% (range 2.1% to 70.4%), with several accessions experiencing 100% disease incidence, which is similar to what was described in commercial fields, where total crop losses were frequently reported every year since 2019 (Hasegawa and Del Pozo-Valdivia, 2022). Our field trials identified accessions with a partial resistance to the disease, such as cvs. Outredgeous Selection (INSVx = -1.27), Red Splash Cos (-1.13), Infantry (-1.11), Sweet Valentine (-1.06), Annapolis 4x (-1.01), Velvet (-1.00), Rubens Red (-0.90), and Eruption (-0.88). When the results of field assessments were compared on 463 accessions evaluated in both the current and the previous study (Simko et al., 2018), a highly significant correlation (*r* = 0.375, *ρ* = 0.365, both *p* < 0.0001) was detected. A relatively high consistency in the results indicate that the same genetic determinants may provide partial resistance to INSV at both periods, regardless of the disease pressure.

Our previous observations from field experiments suggested that lettuce accessions with red color usually show a lower DI% (**Figure 1**). Therefore, we analyzed the content of anthocyanins and chlorophyll, the pigments moderating lettuce leaf color (Simko, 2020), and compared their content to DI%. Both anthocyanins and chlorophyll showed a significant correlation with DI% when tested individually using correlation coefficients or in the multiple linear regression model that considers the combined effect of anthocyanins (ACI-Lb), chlorophyll (SPAD-Sqrt), plant development (PDx), and TFD. While a higher content of anthocyanins correlated negatively (*r* = -0.284, *p* < 0.0001) with DI%, a higher content of chlorophyll correlated (*r* = 0.125, *p* = 0.0053) with increased DI% (**Figure 2**, **Table 2**). A possible relationship between pigments and partial resistance to INSV was also indicated by several loci for disease resistance and anthocyanins or chlorophyll that collocated in the lettuce genome (**Table 4**). It could not be determined from the present results, however, if these pigments play a direct role in resistance to INSV, or if both the resistance and the content of pigments are related to yet another trait. For example, light-green accessions that are more resistant to INSV than dark-green accessions have a lower content of chlorophyll. Because chlorophyll is directly involved in the production of carbohydrates, light-green accessions commonly contain less glucose, fructose, and sucrose (Simko et al., 2014), the compounds that may affect thrips feeding preference (Scott Brown et al., 2002; Pobożniak et al., 2022). Aside from carbohydrates, thrips feeding preference could also be affected by the content of anthocyanins or other physiological factors based on the age of the plant. In onion, a significant positive correlation was described between the abundance of onion thrips (*Thrips tabaci* Lind) and concentrations of total soluble sugars, reducing sugars, and sucrose, while a negative relationship was detected between the total phenolic content and thrips damage (Pobożniak et al., 2022). When greenhouse thrips (*Heliothrips haemorrhoidalis* Bouche) were compared for feeding preferences among several tree species, the levels of soluble proteins present had a greater influence on the susceptibility of the plants than the levels of carbohydrates (Scott Brown et al., 2002). Although these results indicate nutritional and feeding preferences for thrips, it is necessary to evaluate the feeding preferences for western flower thrips on lettuce cultivars due to the potential difference of nutritional requirements between thrips species. We also observed that lettuce accessions that had a faster development rate also had a higher INSV incidence (*r* = 0.352, *p* < 0.0001 between PDx and INSVx). This may be due to differences in nutritional composition, anatomy, phytomorphology, or of some other factors associated with plant development. We cannot exclude a possibility that faster-developing plants had a greater leaf surface area from which thrips fed on.

The lower INSV incidence that was observed for some lettuce accessions may be attributed to thrips feeding preference, plant resistance to thrips, plant resistance to the virus, or a combination of multiple factors. Constitutive plant defenses to thrips include external physical barriers (e.g., trichomes), secondary plant metabolites, stomatal immunity (Arif et al., 2022), or emission of volatile organic compounds (Mouden and Leiss, 2021). Feeding by thrips induces coordinated plant defense strategies, which includes jasmonic acid production (Arif et al., 2022), while the infection by thrips-transmitted viruses triggers the salicylic acid pathway (Mouden and Leiss, 2021). Previous studies reported a strong correlation (*r* = 0.95, *p* = 0.003) between the number of thrips per head of lettuce and INSV incidence when evaluated in six fields of direct-seeded romaine lettuce (Kuo et al., 2014). We have detected a significant, positive correlation between TFD and INSVx on the panel of 496 accessions, although the correlation coefficient was lower (*r* = 0.198, *p* < 0.0001). The significant correlation between TFD and INSVx was confirmed using the multiple linear regression model. When all four evaluated factors (ACI-Lb, SPAD-Sqrt, PDx, and TFD) were used together, the combined model explained 26.5% of the total phenotypic variation in INSVx. The unexplained variation likely includes other factors that contribute to DI%, such as epistatic interaction among loci, variation in inducible plant defense against thrips feeding, and INSV infection. Further research is needed to determine the contributing factors to partial INSV resistance, such as studies on thrips feeding preference (e.g., effect of leaf color or plant composition), plant constitutive resistance (e.g., effect of lettuce head closure and waxy leaf cuticle), mechanisms of induced resistance to thrips, and mechanisms of plant resistance to INSV.

It was previously stated that field evaluations for resistance to INSV are challenging due to the uncertain appearance of the disease and its uneven distribution across a field (Simko et al., 2018). However, in the current study, the frequent and high disease pressure allowed for a more accurate and comprehensive testing of lettuce resistance to INSV in the field. Still, the combined results from multiple field trials (INSVx) seem to provide more reliable results than those from individual field experiments. Such combined data showed comparatively high correlations with all four independent factors (ACI-Lb, SPAD-Sqrt, PDx, and TFD), the highest percentage of explained variation by the multiple linear regression model (**Table 2**), and the largest number of detected QTLs (**Table 3**). It would be desirable to perform more evaluations of TFD, the trait that is likely affected by environmental conditions. However, the time, labor, and experience required to evaluate TFD on the large number of accessions included in the current diversity panel are all limiting factors. Future studies of TFD may include a subset of selected accessions with a broad range of resistance to INSV, which the current study identified. Another complication related to conducting field trials to identify INSV resistance is a possibility of other diseases to be present and co-occur on INSV-infected plants (Smith et al., 2022) (**Figure 1**), thus making the assessment of resistance to INSV infection alone more difficult.

Previous field and greenhouse screenings identified lettuce accessions with partial resistance to INSV (Simko et al., 2018) and a closely related *Tomato spotted wilt virus*—TSWV (O'Malley and Hartmann, 1989), such as cvs. Ancora, Tinto (O'Malley and Hartmann, 1989; Fontes et al., 2019), Amazona, Antigua, Commodore, Eruption, Iceberg, La Brillante, Merlot, and Telluride (Simko et al., 2018). The current study confirmed a relatively high partial resistance in the cultivars that were tested in both this and the previous studies (cvs. Eruption INSVx = -0.88, Iceberg INSVx = -0.64, and La Brillante INSVx = -0.46) (**Supplemental Table 1**). In addition, the current study substantially expanded the list of accessions with partial resistance to INSV, identified the effect of factors (ACI-Lb, SPAD-Sqrt, PDx, and TFD) contributing to DI%, mapped resistance QTLs in the diversity panel and two biparental populations, and identified molecular markers closely linked to the resistance loci. The identified molecular markers can be used to develop assays for marker-assisted selection (MAS) (Simko et al., 2021) or to be used in a combined MAS–genomic selection (Hadasch et al., 2016).

A recent patent describes lettuce genotypes with a monogenic recessive resistance to INSV and TSWV (Schut et al., 2016). Genotypes carrying the genetic determinant (called *insv1* in the current study) in the homozygous state were resistant to both viruses when young lettuce plants were artificially inoculated in a bioassay. Breeding lines with the *insv1* resistance gene showed a substantial reduction in DI% when tested in field conditions in the Salinas Valley, although they were not completely resistant to the virus. INSV DI% in the breeding lines ranged from 0.4% to 7.0% in three field experiments, while in control cultivars, the DI% was in the range from 10.9% to 41.2% (Smith et al., 2022). The *insv1* gene is located on chr. 2 (**Figure 4**). It seems to be separated by ca. 26 Mbp from *qINSV2.1*, the QTL for INSV resistance that was the most frequently detected QTL in the current study. The genomic location and the phenotypic effect of the *qINSV2.1* was independently confirmed in another biparental mapping population (Richardson, Nayak, and Simko, unpublished results). Field and laboratory studies are underway to dissect a complex interplay between lettuce, thrips, and resistance to INSV.

## Data availability statement

The original contributions presented in the study are included in the article/**Supplementary Material**. Further inquiries can be directed to the corresponding author.

## Author contributions

IS: Conceptualization, Investigation, Funding acquisition, Supervision, Project administration, Methodology, Formal analysis, Writing—Original Draft, and Writing—Review and Editing. DH: Investigation, Funding acquisition, and Writing—Review and Editing. HP: Formal analysis and Writing—Review and Editing. RZ: Investigation and Writing—Review and Editing. All authors contributed to the article and approved the submitted version.

## Supplementary material

Supplementary Table 1 Phenotypic data for 496 accessions used in GWAS (plus two *L. indica* accessions). Data are provided for INSV incidence, content of chlorophyll and anthocyanins, plant development, and thrips feeding damage.

Supplementary Figure 1 Lettuce INSV incidence in 12 field experiments and combined data (INSVx) across experiments. Green histograms in diagonal position illustrate distributions of disease incidence (data were transformed using the bestNormalize R package (Peterson and Cavanaugh, 2020) for each experiment. Values above histograms correlation coefficients between disease incidence data from 13 datasets. Color and size of the circles indicate the size of the correlation coefficients and their significance as shown in the legend. Plots below histograms depict linear fit between disease incidence data from 13 datasets (black line) and its 95% confidence interval (red shaded area). Nonparametric density of data is indicated by gray (lower density) and red (higher density) areas.
