# Supplementary figures and images for: Genetic and physiological determinants of lettuce partial resistance to Impatiens necrotic spot virus
Source: Front Plant Sci. 2023 Jun 8;14:1163683. doi: 10.3389/fpls.2023.1163683 (PMC10285314; doi:10.3389/fpls.2023.1163683)

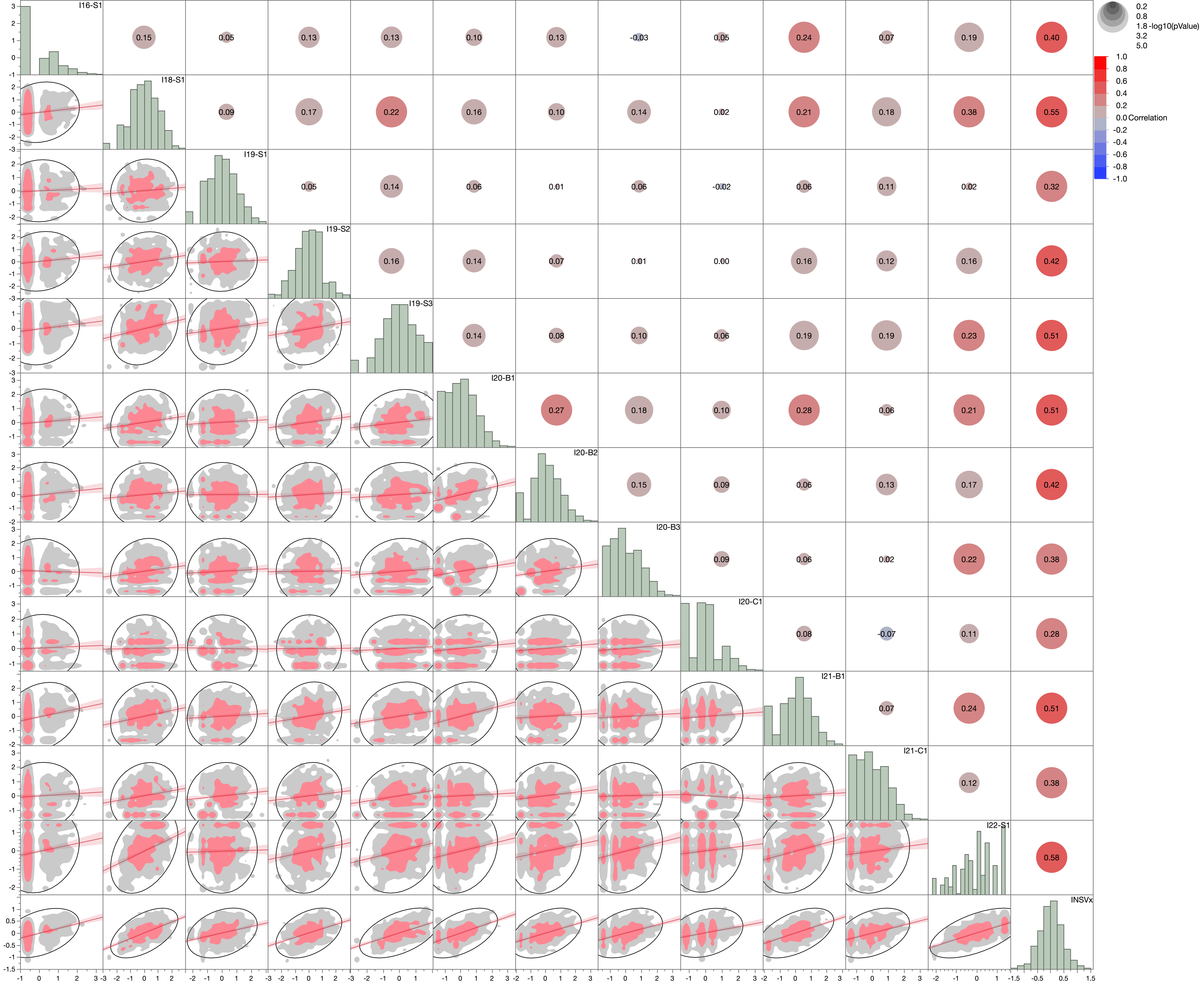

Supplement: Supplementary Figure 1 — Lettuce INSV incidence in 12 field experiments and combined data (INSVx) across experiments. Green histograms in diagonal position illustrate distributions of disease incidence (data were transformed using the bestNormalize R package (Peterson and Cavanaugh, 2020) for each experiment. Values above histograms correlation coefficients between disease incidence data from 13 datasets. Color and size of the circles indicate the size of the correlation coefficients and their significance as shown in the legend. Plots below histograms depict linear fit between disease incidence data from 13 datasets (black line) and its 95% confidence interval (red shaded area). Nonparametric density of data is indicated by gray (lower density) and red (higher density) areas. [file DataSheet_1.pdf]
